# Crystal structure and Hirshfeld surface analysis of *N*-(1*H*-benzo[*d*]imidazol-2-yl)acetamide

**DOI:** 10.1107/S2056989026004196

**Published:** 2026-04-29

**Authors:** Akmaljon G. Tojiboev, Rasul Okmanov, Asqar Abdurazakov, Sarvar Saidov, Elyor Rakhmatov, Abduakhad Kodirov, Burkhon Elmuradov

**Affiliations:** a University of Geological Sciences, Olimlar str. 64, Tashkent 100170, Uzbekistan; b Institute of the Chemistry of Plant Substances, Uzbekistan Academy of Sciences, Mirzo Ulugbek Str. 77, Tashkent 100170, Uzbekistan; c Karshi State University, Kuchabog str. 17, Karshi 180119, Uzbekistan; Vienna University of Technology, Austria

**Keywords:** hy­dro­gen bonding, crystal structure, Hirshfeld surface analysis, benzimidazole

## Abstract

The crystal structure of the title com­pound, C_9_H_9_N_3_O, was refined using non-spherical scattering factors. This quantum crystallographic approach provided enhanced precision for the H-atom positions and a refined description of the electron density. The asymmetric unit com­prises two mol­ecules (*Z*′ = 2) exhibiting high conformational similarity (r.m.s. deviation between the mol­ecules is 0.083 Å). In the crystal, mol­ecules form pseudocentrosymmetric dimers *via* inter­molecular N—H⋯O and N—H⋯N hy­dro­gen bonds. These units are further linked into supra­molecular layers characterized by *D*(1), *C*_2_^2^(8), *C*_2_^2^(10), *R*_2_^2^(8) and *R*_2_^2^(12) graph-set motifs. The packing is mainly consolidated by C—H⋯π inter­actions. Hirshfeld surface analysis and two-dimensional fingerprint plots were used to qu­antify the supra­molecular assembly, identifying H⋯H (45%), C⋯H/H⋯C (20.8%), N⋯H/H⋯N (12.2%) and O⋯H/H⋯O (11.5%) contacts as the primary contributors to the crystal packing.

## Abstract 2

The crystal structure of *N*-(1*H*-benzo[*d*]imidazol-2-yl)acetamide, refined using non-spherical atomic scattering factors, features a pseudocentrosymmetric dimeric assembly governed by N—H⋯O and N—H⋯N hy­dro­gen bonds, with its supra­molecular assembly further consolidated by C—H⋯π inter­actions, as qu­anti­fied by Hirshfeld surface analysis.

## 1. Chemical context

Benzimidazole is a bicyclic heteroaromatic organic com­pound consisting of a benzene ring and an imidazole ring, which enables chemists to carry out targeted electrophilic and nucleophilic substitution or addition reactions (Faheem *et al.*, 2020 ▸). Substituted benzimidazoles constitute an important class of heterocyclic com­pounds that are of inter­est to both theoretical organic chemists and representatives of the pharmaceutical industry (Lee *et al.*, 2023 ▸), in particular due to their anti­microbial, anthelmintic, anti­viral and anti­cancer activities, or their use as anti­hypertensives and anti­histamines, *e.g.* astemizole or bilastine (Chung *et al.*, 2023 ▸).
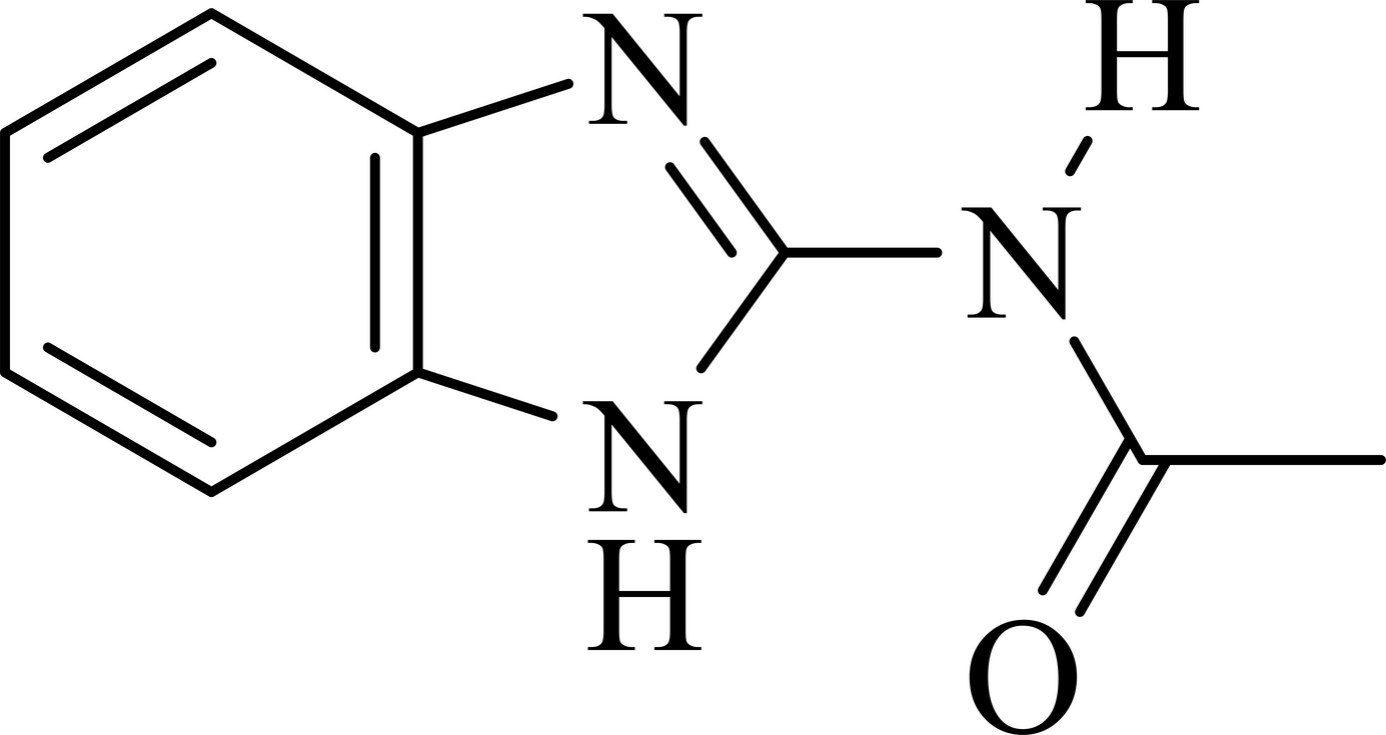


In this context, we report the synthesis and crystal structure determination of *N*-(1*H*-benzo[*d*]imidazol-2-yl)acetamide, (**1**), and provide the results of a Hirshfeld surface analysis.

## 2. Structural commentary

**Figure 1:**
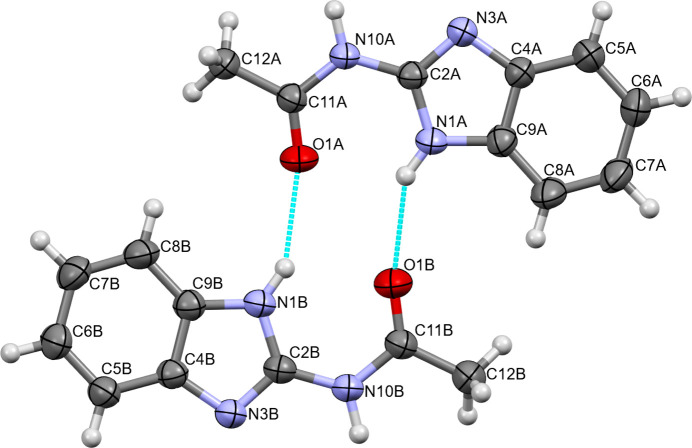
The structures of independent mol­ecules *A* and *B* in com­pound (**1**), with the atom-labeling scheme and displacement ellipsoids drawn at the 50% probability level (H atoms are shown as spheres of arbitrary size). Inter­molecular N1*A*—H1*A*⋯O1*B* and N1*B*—H1*B*⋯O1*A* hy­dro­gen bonds are shown as blue dashed lines.

**Figure 2:**
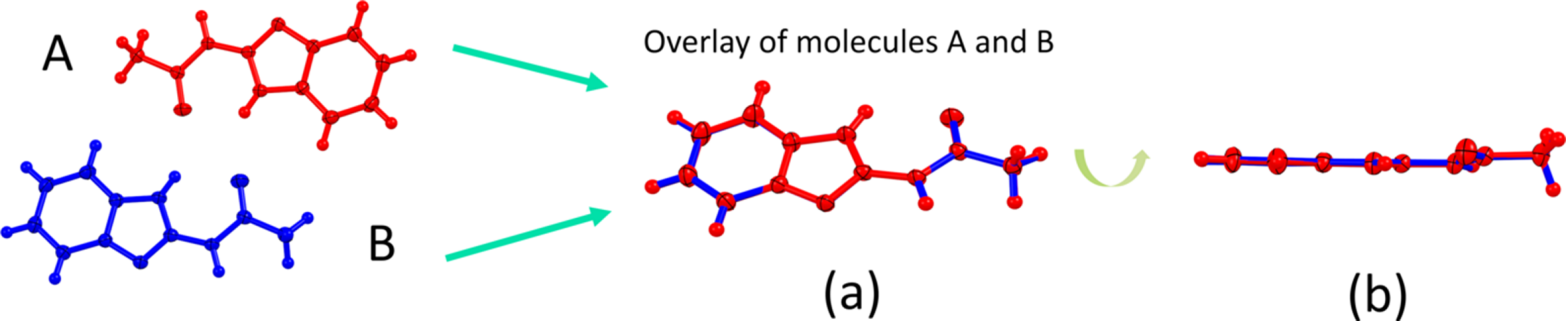
(*a*) Superposition of mol­ecule *A* (red) and mol­ecule *B* (blue) in the title com­pound, and (*b*) side views of mol­ecules *A* and *B* to show their planarity.

The asymmetric unit of com­pound (**1**) com­prises two mol­ecules designated as *A* and *B* (Fig. 1 ▸). Mol­ecules *A* and *B* form a pseudocentrosymmetric dimer consolidated by two non-equivalent inter­molecular N1*A*—H1*A*⋯O1*B* and N1*B*—H1*B*⋯O1*A* hy­dro­gen bonds (Table 1 ▸; entries 1 and 2). In each case, the mol­ecular structure is stabilized by an intra­molecular N—H⋯O hy­dro­gen bond (Table 1 ▸; entries 3 and 4), which leads to the formation of a five-membered ring. The r.m.s. deviation between the non-H atoms of mol­ecules *A* and *B* is 0.083 Å, indicating a high degree of structural similarity (Fig. 2 ▸). Both mol­ecules are essentially planar, with r.m.s. deviations of 0.0123 (for *A*) and 0.0122 Å (for *B*). The planarity is further supported by torsion angles C11—N10—C2—N3 = 170.41 (11)° for *A* and −175.67 (12)° for *B*, which deviate only slightly from the ideal anti­periplanar value of 180°. The observed planarity facilitates maximum π-electron conjugation and *p*-orbital overlap, providing the mol­ecular framework with substantial electronic stability.

## 3. Supra­molecular features

**Figure 3:**
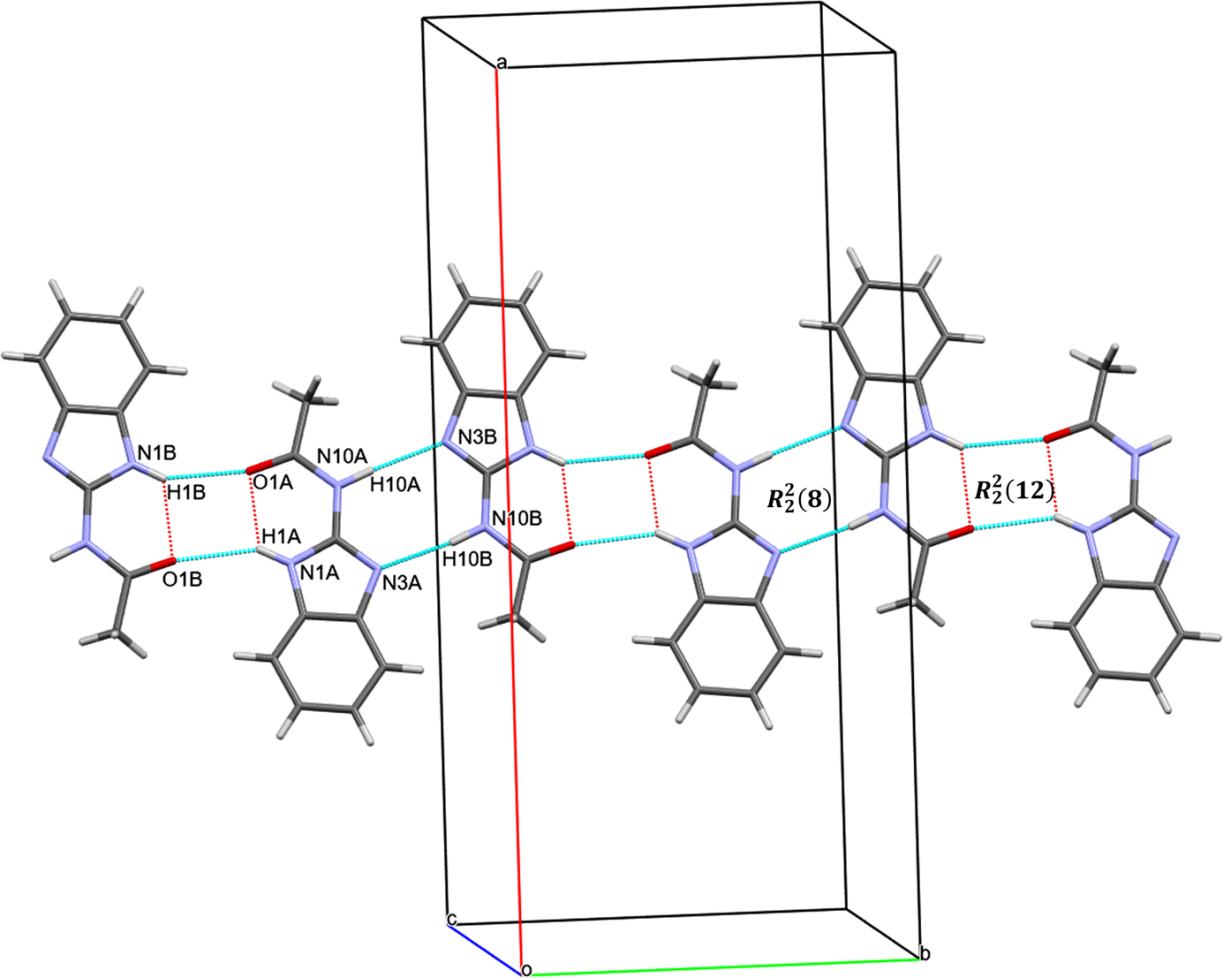
Intra­molecular N1*B*—H1*B*⋯O1*B* hy­dro­gen bonds (red dashed lines) and inter­molecular N1*B*—H1*B*⋯O1*A*, N1*A*—H1*A*⋯O1*B*, N10*A*—H10*A*⋯N3*B*, N10*B*—H10*B*⋯N3*A* hy­dro­gen bonds (blue dashed lines). The 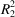
(8) and 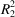
(12) graph-set motifs, extending parallel to the *b* axis, are generated by a combination of N—H⋯N and N—H⋯O hy­dro­gen bonds.

In the crystal, the inter­play between intra- and inter­molecular N—H⋯O hy­dro­gen bonds (Table 1 ▸, entries 1–4), as well as of inter­molecular N—H⋯N hy­dro­gen bonds (Table 1 ▸; entries 5 and 6), leads to the formation of chains propagating parallel to the *b* axis (Fig. 3 ▸) and gives rise to *D*(1), *C*_2_^2^(8), *C*_2_^2^(10), *R*_2_^2^(8) and 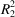
(12) graph-set motifs (Etter *et al.*, 1990 ▸). The mol­ecules in adjacent chains inter­act mainly through C—H⋯π inter­<!?tlsb=-0.05pt>actions, specifically C12*A*—H12*B*⋯*Cg*2 [3.523 (2) Å], C12*B*—H12*E*⋯*Cg*4 [3.526 (2) Å] and C12*B*—H12*F*⋯*Cg*5 [3.526 (2) Å] [*Cg*2 is the centroid of ring C4*A*–C9*A*, *Cg*4 is the centroid of ring N1*B*/C2*B*/N3*B*/C4*B*/C9*B* and *Cg*5 is the centroid of ring C4*B*–C9*B*] (Fig. S1 in the supporting information). These inter­actions contribute to a herringbone packing motif in the crystal structure (Fig. S2).

## 4. Database survey

A search of the Cambridge Structural Database (CSD, Version 2025.3.0; Groom *et al.*, 2016 ▸) for structures containing the 2-acetamido­benzimidazole moiety with similar planarity yielded ten relevant hits. These include refcodes BELYEA (Bhardwaj *et al.*, 2022 ▸), FIZSUF (Odame *et al.*, 2018 ▸), LUMCAA (Gergely *et al.*, 2020 ▸), PUPJIW (Al-Taie *et al.*, 2020 ▸), PUPJOC (Al-Taie *et al.*, 2020 ▸), PUPJUI (Al-Taie *et al.*, 2020 ▸), SOVZAH (Srinivasarao *et al.*, 2019 ▸), VADKIY (Singh *et al.*, 2017 ▸), WEDJIC (Kumari *et al.*, 2022 ▸) and XENKAD (Yang *et al.*, 2006 ▸). As already noted, com­pound (**1**) exhibits a high degree of planarity, which is a common feature among the surveyed structures. However, significant differences arise in the orientation of the acetamide substituent. The conformation is primarily governed by the C11—N10—C2—N3 torsion angle. Mol­ecule *A* of the title com­pound shows a nearly coplanar arrangement, closely resembling the conformations found in LUMCAA and XENKAD. In contrast, mol­ecule *B* exhibits a slight twist that correlates more closely with the mol­ecular shape of FIZSUF. Furthermore, the CSD survey reveals two com­peting hy­dro­gen-bonding motifs for this class of com­pounds. While many derivatives, such as PUPJIW and SOVZAH, favour the formation of centrosymmetric 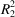
(8) dimers, the title com­pound utilizes both mol­ecules *A* and *B* to establish a pseudocentrosymmetric dimer through N—H⋯O inter­actions. This specific assembly is influenced by the steric requirements of the benzimidazole core, distinguishing it from the simpler chain motifs as observed, for example, in VADKIY.

## 5. Hirshfeld surface analysis

**Figure 4:**
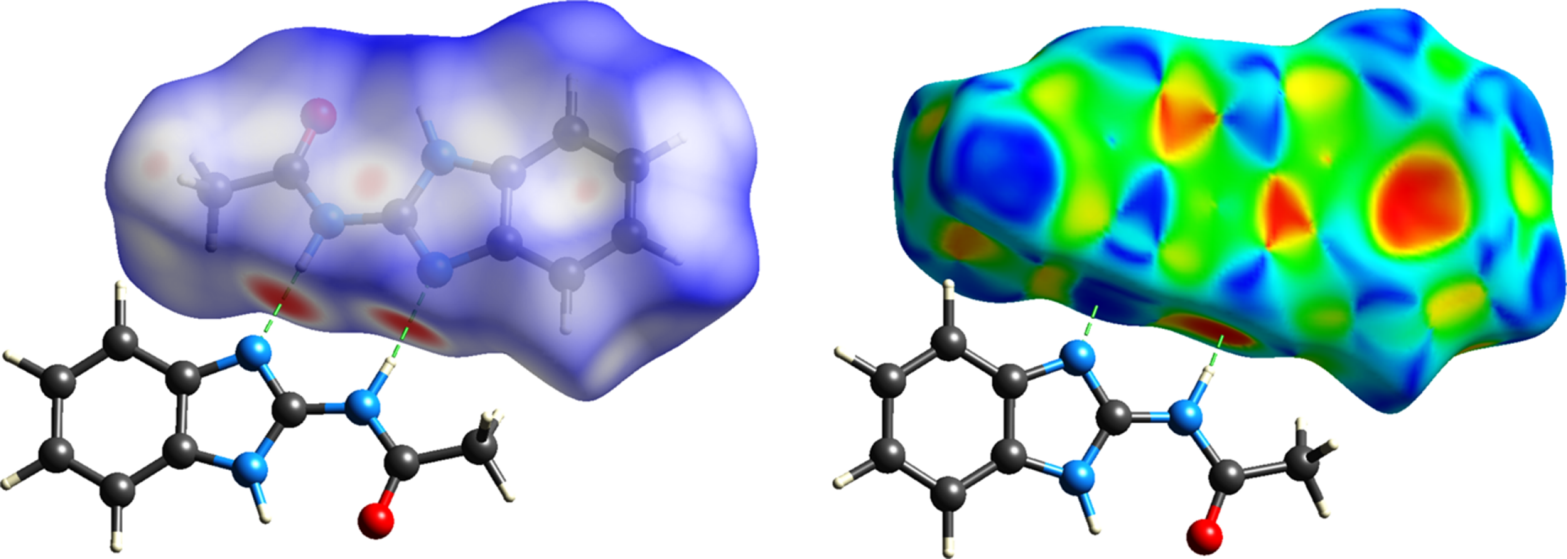
Hirshfeld surface of (**1**), mapped over *d*_norm_ (left) and shape index (right), showing close inter­molecular con­tacts.

**Figure 5:**
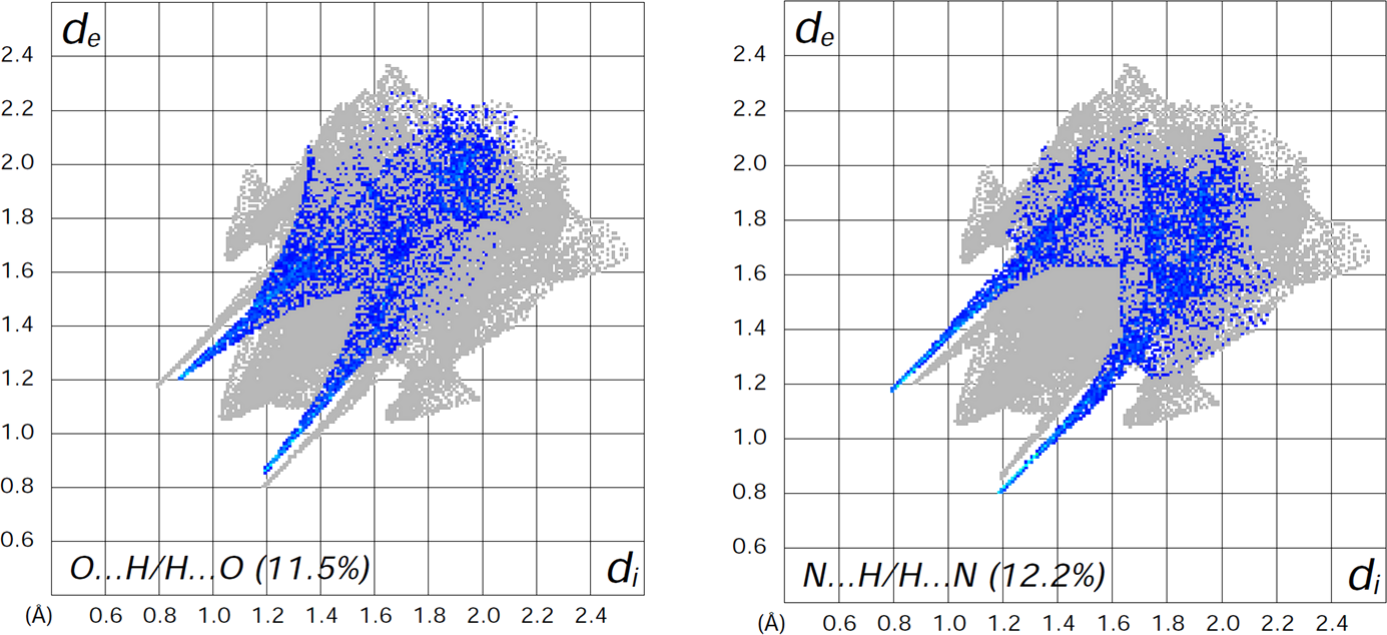
Two-dimensional fingerprint plots for the title com­pound, decom­posed into (left) O⋯H/H⋯O (11.5%) and (right) N⋯H/H⋯N (12.2%) contacts.

To gain deeper insight into the inter­molecular inter­actions within the title com­pound, a Hirshfeld surface (HS) analysis was carried out, and two-dimensional fingerprint plots were generated using *CrystalExplorer* (Spackman *et al.*, 2021 ▸). The HS mapped over *d*_norm_, and the shape index as a visual representation of the contacts, are shown in Fig. 4 ▸. The *d*_norm_ surface exhibits prominent deep-red spots, which correspond to the closest contact distances, specifically representing the donor and acceptor sites of the inter­molecular N—H⋯O and N—H⋯N hy­dro­gen bonds. The relative contributions of the various inter­molecular contacts were qu­anti­fied using two-dimensional fingerprint plots (Fig. S3 in the supporting information), revealing that the stability of the crystal packing is primarily governed by H⋯H contacts, which constitute the largest contribution at 45.0%. The significant role of C—H⋯π inter­actions is evidenced by the C⋯H/H⋯C contacts (20.8%). The presence of classical hy­dro­gen bonding is clearly manifested as a pair of characteristic sharp ‘spikes’ in the fingerprint plots (Fig. 5 ▸) for N—H⋯N inter­actions (repre­sent­ed by N⋯H/H⋯N contacts, 12.2%) and N—H⋯O amide inter­actions (repre­sent­ed by O⋯H/H⋯O contacts, 11.5%). A detailed inspection of the shape index map reveals a pattern of red and blue triangles (‘bow-tie’ patterns) and com­plementary flat regions on the curvedness map. These features, combined with the C⋯C contribution (2.0%), are indicative of weak π–π stacking inter­actions between the benzimidazole rings. Minor contributions from C⋯O/O⋯C (3.1%), N⋯C/C⋯N (2.2%) and other contacts (totaling approximately 3.2%) further facilitate the supra­molecular assembly in the crystal.

## 6. Synthesis and crystallization

**Figure 6:**
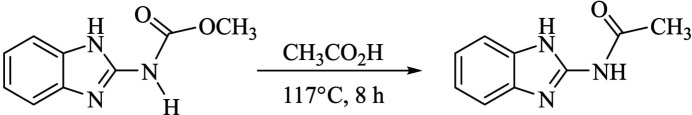
Synthesis scheme to obtain the title com­pound.

The reaction of methyl­benzimidazol-2-yl carbamate with glacial acetic acid was carried out at the boiling point of the acid for 8 h. As a result of the reaction, *N*-(1*H*-benzimidazol-2-yl)acetamide was synthesized (Fig. 6 ▸) in almost qu­anti­tative yield (Abduraza­kov *et al.*, 2021 ▸). Colourless single crystals suitable for X-ray diffraction analysis were obtained by recrystallization from methanol.

## 7. Refinement

Crystal data, data collection and structure refinement details are summarized in Table 2 ▸. H atoms were first positioned geometrically [aromatic C—H = 0.93 Å and N—H = 0.86 Å, and in the acetamido fragment N—H = 0.924 (16) Å] and refined using a riding model, with *U*_iso_(H) = 1.2*U*_eq_(aromatic C, N) or 1.5*U*_eq_(methyl C). The structure was finally refined using the *NoSpherA2* (Kleemiss *et al.*, 2021 ▸) implementation within *OLEX2* (Bourhis *et al.*, 2015 ▸). Non-spherical atomic scattering factors were calculated using the *ORCA* (Neese, 2012 ▸) software package at the r2SCAN/3-21G (Furness *et al.*, 2020 ▸) level of theory. The RIJCOSX approximation with the def2/J auxiliary basis set was employed to accelerate the calculation of the electronic structure. This approach allowed for a more accurate treatment of the electron density, particularly for H-atom positions and the resulting inter­molecular inter­actions.

## Supplementary Material

Crystal structure: contains datablock(s) I. DOI: 10.1107/S2056989026004196/wm5794sup1.cif

Figures S1, S2, and S3 with their captions. DOI: 10.1107/S2056989026004196/wm5794sup2.pdf

Supporting information file. DOI: 10.1107/S2056989026004196/wm5794Isup3.cml

CCDC reference: 2394740

Additional supporting information:  crystallographic information; 3D view; checkCIF report

## Figures and Tables

**Table 1:** Hydrogen-bond geometry (Å, °)

*D*—H⋯*A*	*D*—H	H⋯*A*	*D*⋯*A*	*D*—H⋯*A*
N1*A*—H1*A*⋯O1*B*	1.03 (1)	2.06 (1)	2.9647 (15)	145 (1)
N1*B*—H1*B*⋯O1*A*	1.03 (1)	2.05 (1)	2.9693 (15)	148 (1)
N1*A*—H1*A*⋯O1*A*	1.03 (1)	2.10 (1)	2.6780 (16)	114 (1)
N1*B*—H1*B*⋯O1*B*	1.03 (1)	2.10 (1)	2.6822 (17)	114 (1)
N10*A*—H10*A*⋯N3*B*^i^	1.031 (15)	1.948 (15)	2.9757 (16)	174.7 (13)
N10*B*—H10*B*⋯N3*A*^ii^	1.026 (14)	1.962 (14)	2.9837 (16)	173.7 (11)

Symmetry codes: (i) 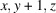; (ii) 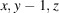.

**Table 2:** Experimental details

Crystal data	
Chemical formula	C_9_H_9_N_3_O
*M*_r_	175.19
Crystal system, space group	Orthorhombic, *Pbcn*
Temperature (K)	293
*a*, *b*, *c* (Å)	23.011 (5), 10.167 (2), 14.294 (3)
*V* (Å^3^)	3344.0 (12)
*Z*	16
Radiation type	Cu *K*α
μ (mm^−1^)	0.79
Crystal size (mm)	0.30 × 0.25 × 0.25

Data collection	
Diffractometer	XtaLAB Synergy, Single source at home/near, HyPix3000
Absorption correction	Multi-scan (*CrysAlis PRO*; Rigaku OD, 2020 ▸)
*T*_min_, *T*_max_	0.906, 1.000
No. of measured, independent and observed [*I* ≥ 2u(*I*)] reflections	32896, 3484, 2875
*R*_int_	0.050
(sin θ/λ)_max_ (Å^−1^)	0.629

Refinement	
*R*[*F*^2^ > 2σ(*F*^2^)], *wR*(*F*^2^), *S*	0.035, 0.103, 1.05
No. of reflections	3484
No. of parameters	246
H-atom treatment	H atoms treated by a mixture of independent and constrained refinement
Δρ_max_, Δρ_min_ (e Å^−3^)	0.23, −0.25

Computer programs: *CrysAlis PRO* (Rigaku OD, 2020 ▸), *SHELXT* (Sheldrick, 2015 ▸), *OLEX2*.refine (Bourhis *et al.*, 2015 ▸), *OLEX2* (Dolomanov *et al.*, 2009 ▸) and *publCIF* (Westrip, 2010 ▸).

## supplementary crystallographic information

### N-(1H-Benzo[d]imidazol-2-yl)acetamide Crystal data

**Table:**

C_9_H_9_N_3_O	*D*_x_ = 1.392 Mg m^−^^3^
*M**_r_* = 175.19	Cu *K*α radiation, λ = 1.54184 Å
Orthorhombic, *P**b**c**n*	Cell parameters from 9360 reflections
*a* = 23.011 (5) Å	θ = 4.3–75.7°
*b* = 10.167 (2) Å	µ = 0.79 mm^−^^1^
*c* = 14.294 (3) Å	*T* = 293 K
*V* = 3344.0 (12) Å^3^	Prism, colourless
*Z* = 16	0.30 × 0.25 × 0.25 mm
*F*(000) = 1477.168	

### N-(1H-Benzo[d]imidazol-2-yl)acetamide Data collection

**Table:**

XtaLAB Synergy, Single source at home/near, HyPix3000 diffractometer	3484 independent reflections
Radiation source: fine-focus sealed X-ray tube, Enhance (Cu) X-ray Source	2875 reflections with *I*≥ 2u(*I*)
Graphite monochromator	*R*_int_ = 0.050
Detector resolution: 10.2576 pixels mm^-1^	θ_max_ = 76.0°, θ_min_ = 3.8°
ω scans	*h* = −28→28
Absorption correction: multi-scan (CrysAlis PRO; Rigaku OD, 2020)	*k* = −11→12
*T*_min_ = 0.906, *T*_max_ = 1.000	*l* = −12→17
32896 measured reflections	

### N-(1H-Benzo[d]imidazol-2-yl)acetamide Refinement

**Table:**

Refinement on *F*^2^	0 restraints
Least-squares matrix: full	28 constraints
*R*[*F*^2^ > 2σ(*F*^2^)] = 0.035	H atoms treated by a mixture of independent and constrained refinement
*wR*(*F*^2^) = 0.103	*w* = 1/[σ^2^(*F*_o_^2^) + (0.0545*P*)^2^ + 0.3951*P*] where *P* = (*F*_o_^2^ + 2*F*_c_^2^)/3
*S* = 1.05	(Δ/σ)_max_ = 0.001
3484 reflections	Δρ_max_ = 0.23 e Å^−^^3^
246 parameters	Δρ_min_ = −0.25 e Å^−^^3^

### N-(1H-Benzo[d]imidazol-2-yl)acetamide Fractional atomic coordinates and isotropic or equivalent isotropic displacement parameters (Å^2^)

**Table:**

	*x*	*y*	*z*	*U*_iso_*/*U*_eq_	
O1A	0.54314 (4)	0.42827 (9)	0.41538 (7)	0.0506 (3)	
N1A	0.44426 (5)	0.51032 (10)	0.33566 (8)	0.0410 (3)	
H1A	0.46369 (5)	0.42009 (10)	0.34373 (8)	0.0492 (3)*	
C2A	0.46844 (5)	0.62938 (11)	0.35331 (8)	0.0347 (3)	
N3A	0.43391 (4)	0.72967 (10)	0.33542 (7)	0.0395 (2)	
C4A	0.38259 (5)	0.67161 (12)	0.30455 (9)	0.0388 (3)	
C5A	0.32989 (6)	0.72712 (14)	0.27686 (10)	0.0512 (3)	
H5A	0.32414 (6)	0.83284 (14)	0.27590 (10)	0.0614 (4)*	
C6A	0.28500 (6)	0.64385 (15)	0.25056 (11)	0.0544 (4)	
H6A	0.24381 (6)	0.68572 (15)	0.22918 (11)	0.0653 (4)*	
C7A	0.29165 (6)	0.50760 (16)	0.25101 (12)	0.0568 (4)	
H7A	0.25558 (6)	0.44570 (16)	0.23024 (12)	0.0681 (5)*	
C8A	0.34374 (6)	0.45002 (14)	0.27762 (11)	0.0553 (4)	
H8A	0.34941 (6)	0.34427 (14)	0.27735 (11)	0.0664 (5)*	
C9A	0.38835 (5)	0.53409 (13)	0.30468 (9)	0.0400 (3)	
N10A	0.52424 (4)	0.64415 (10)	0.38705 (7)	0.0370 (2)	
H10A	0.5399 (7)	0.7384 (15)	0.3954 (10)	0.042 (4)*	
C11A	0.55827 (5)	0.54332 (12)	0.41863 (9)	0.0375 (3)	
C12A	0.61590 (5)	0.58512 (13)	0.45685 (10)	0.0447 (3)	
H12A	0.6338 (2)	0.5070 (4)	0.4988 (6)	0.0670 (5)*	
H12B	0.64500 (14)	0.6068 (9)	0.39989 (10)	0.0670 (5)*	
H12C	0.61036 (8)	0.6717 (6)	0.4994 (6)	0.0670 (5)*	
O1B	0.44986 (5)	0.22170 (9)	0.36385 (8)	0.0588 (3)	
N1B	0.55554 (5)	0.13757 (10)	0.41569 (8)	0.0421 (3)	
H1B	0.53532 (5)	0.22762 (10)	0.41509 (8)	0.0505 (3)*	
C2B	0.53160 (5)	0.02000 (12)	0.39257 (8)	0.0364 (3)	
N3B	0.56756 (5)	−0.08061 (10)	0.39924 (8)	0.0392 (2)	
C4B	0.61967 (5)	−0.02432 (12)	0.42884 (9)	0.0374 (3)	
C5B	0.67373 (6)	−0.08151 (13)	0.44646 (10)	0.0466 (3)	
H5B	0.68069 (6)	−0.18583 (13)	0.43602 (10)	0.0559 (4)*	
C6B	0.71827 (6)	−0.00072 (14)	0.47777 (11)	0.0522 (4)	
H6B	0.76041 (6)	−0.04342 (14)	0.49250 (11)	0.0626 (4)*	
C7B	0.71020 (6)	0.13435 (15)	0.49078 (11)	0.0543 (4)	
H7B	0.74578 (6)	0.19383 (15)	0.51665 (11)	0.0651 (4)*	
C8B	0.65723 (6)	0.19316 (14)	0.47100 (11)	0.0518 (3)	
H8B	0.65092 (6)	0.29809 (14)	0.47923 (11)	0.0621 (4)*	
C9B	0.61259 (5)	0.11201 (12)	0.44021 (9)	0.0398 (3)	
N10B	0.47449 (5)	0.00536 (10)	0.36460 (8)	0.0391 (2)	
H10B	0.4577 (6)	−0.0867 (14)	0.3530 (9)	0.035 (3)*	
C11B	0.43657 (6)	0.10695 (12)	0.35088 (9)	0.0409 (3)	
C12B	0.37728 (6)	0.06656 (14)	0.31914 (11)	0.0511 (3)	
H12D	0.34675 (10)	0.1440 (5)	0.3343 (7)	0.0767 (5)*	
H12E	0.37793 (12)	0.0484 (10)	0.24493 (17)	0.0767 (5)*	
H12F	0.3645 (2)	−0.0217 (6)	0.3553 (6)	0.0767 (5)*	

### N-(1H-Benzo[d]imidazol-2-yl)acetamide Atomic displacement parameters (Å^2^)

**Table:**

	*U*^11^	*U*^22^	*U*^33^	*U*^12^	*U*^13^	*U*^23^
O1A	0.0473 (5)	0.0266 (5)	0.0779 (7)	−0.0012 (4)	−0.0113 (5)	0.0061 (4)
N1A	0.0401 (6)	0.0268 (5)	0.0562 (6)	−0.0025 (4)	−0.0057 (5)	0.0044 (4)
C2A	0.0353 (6)	0.0264 (6)	0.0425 (6)	−0.0005 (4)	−0.0006 (5)	0.0012 (4)
N3A	0.0348 (5)	0.0287 (5)	0.0550 (6)	0.0016 (4)	−0.0055 (4)	−0.0033 (4)
C4A	0.0335 (6)	0.0346 (7)	0.0483 (6)	0.0001 (5)	−0.0025 (5)	−0.0012 (5)
C5A	0.0385 (7)	0.0416 (8)	0.0734 (9)	0.0036 (6)	−0.0100 (6)	−0.0063 (6)
C6A	0.0346 (7)	0.0553 (9)	0.0733 (9)	−0.0007 (6)	−0.0091 (6)	−0.0003 (7)
C7A	0.0411 (8)	0.0521 (9)	0.0771 (10)	−0.0162 (6)	−0.0132 (7)	0.0086 (7)
C8A	0.0502 (8)	0.0379 (8)	0.0778 (10)	−0.0123 (6)	−0.0158 (7)	0.0113 (7)
C9A	0.0362 (6)	0.0354 (7)	0.0484 (7)	−0.0041 (5)	−0.0036 (5)	0.0054 (5)
N10A	0.0338 (5)	0.0265 (5)	0.0505 (6)	−0.0005 (4)	−0.0030 (4)	0.0018 (4)
C11A	0.0343 (6)	0.0298 (6)	0.0483 (6)	−0.0001 (5)	−0.0015 (5)	0.0032 (5)
C12A	0.0376 (7)	0.0381 (7)	0.0584 (8)	−0.0004 (5)	−0.0061 (5)	0.0033 (6)
O1B	0.0508 (6)	0.0281 (5)	0.0976 (8)	0.0014 (4)	−0.0140 (5)	0.0000 (5)
N1B	0.0411 (6)	0.0272 (5)	0.0579 (6)	0.0005 (4)	−0.0015 (5)	−0.0021 (4)
C2B	0.0390 (6)	0.0270 (6)	0.0433 (6)	0.0005 (5)	−0.0006 (5)	−0.0003 (5)
N3B	0.0374 (5)	0.0275 (5)	0.0527 (6)	0.0006 (4)	−0.0021 (4)	−0.0037 (4)
C4B	0.0352 (6)	0.0315 (6)	0.0456 (6)	−0.0012 (5)	0.0024 (5)	−0.0018 (5)
C5B	0.0369 (7)	0.0370 (7)	0.0659 (8)	0.0011 (5)	0.0006 (6)	−0.0020 (6)
C6B	0.0346 (7)	0.0476 (8)	0.0743 (9)	−0.0032 (6)	0.0015 (6)	−0.0008 (7)
C7B	0.0378 (7)	0.0479 (8)	0.0771 (9)	−0.0102 (6)	0.0006 (7)	−0.0056 (7)
C8B	0.0448 (8)	0.0348 (7)	0.0757 (9)	−0.0068 (6)	−0.0003 (7)	−0.0066 (6)
C9B	0.0386 (6)	0.0319 (6)	0.0489 (7)	−0.0023 (5)	0.0008 (5)	−0.0018 (5)
N10B	0.0378 (5)	0.0288 (5)	0.0509 (6)	0.0007 (4)	−0.0035 (4)	−0.0024 (4)
C11B	0.0403 (7)	0.0297 (6)	0.0527 (7)	0.0020 (5)	−0.0027 (5)	0.0009 (5)
C12B	0.0435 (7)	0.0410 (8)	0.0688 (9)	0.0024 (6)	−0.0086 (6)	−0.0020 (6)

### N-(1H-Benzo[d]imidazol-2-yl)acetamide Geometric parameters (Å, º)

**Table:**

O1A—C11A	1.2212 (15)	O1B—C11B	1.2202 (16)
N1A—H1A	1.0270	N1B—H1B	1.0270
N1A—C2A	1.3560 (15)	N1B—C2B	1.3569 (16)
N1A—C9A	1.3818 (16)	N1B—C9B	1.3835 (16)
C2A—N3A	1.3177 (15)	C2B—N3B	1.3190 (15)
C2A—N10A	1.3797 (16)	C2B—N10B	1.3818 (16)
N3A—C4A	1.3920 (15)	N3B—C4B	1.3945 (16)
C4A—C5A	1.3951 (17)	C4B—C5B	1.3960 (17)
C4A—C9A	1.4043 (18)	C4B—C9B	1.4050 (17)
C5A—H5A	1.0830	C5B—H5B	1.0830
C5A—C6A	1.3874 (19)	C5B—C6B	1.3876 (18)
C6A—H6A	1.0830	C6B—H6B	1.0830
C6A—C7A	1.394 (2)	C6B—C7B	1.398 (2)
C7A—H7A	1.0830	C7B—H7B	1.0830
C7A—C8A	1.387 (2)	C7B—C8B	1.387 (2)
C8A—H8A	1.0830	C8B—H8B	1.0830
C8A—C9A	1.3908 (18)	C8B—C9B	1.3890 (18)
N10A—H10A	1.031 (15)	N10B—H10B	1.026 (14)
N10A—C11A	1.3667 (15)	N10B—C11B	1.3661 (16)
C11A—C12A	1.4957 (17)	C11B—C12B	1.4953 (18)
C12A—H12A	1.0770	C12B—H12D	1.0770
C12A—H12B	1.0770	C12B—H12E	1.0770
C12A—H12C	1.0770	C12B—H12F	1.0770
			
C2A—N1A—H1A	126.71 (7)	C2B—N1B—H1B	126.83 (7)
C9A—N1A—H1A	126.71 (7)	C9B—N1B—H1B	126.83 (7)
C9A—N1A—C2A	106.59 (10)	C9B—N1B—C2B	106.35 (10)
N3A—C2A—N1A	114.03 (11)	N3B—C2B—N1B	114.25 (11)
N10A—C2A—N1A	122.96 (11)	N10B—C2B—N1B	123.47 (11)
N10A—C2A—N3A	123.01 (10)	N10B—C2B—N3B	122.28 (11)
C4A—N3A—C2A	104.17 (10)	C4B—N3B—C2B	104.08 (10)
C5A—C4A—N3A	130.99 (12)	C5B—C4B—N3B	130.55 (12)
C9A—C4A—N3A	109.98 (11)	C9B—C4B—N3B	109.89 (11)
C9A—C4A—C5A	119.02 (12)	C9B—C4B—C5B	119.56 (12)
H5A—C5A—C4A	120.75 (8)	H5B—C5B—C4B	120.99 (8)
C6A—C5A—C4A	118.51 (13)	C6B—C5B—C4B	118.03 (12)
C6A—C5A—H5A	120.75 (9)	C6B—C5B—H5B	120.99 (8)
H6A—C6A—C5A	119.22 (9)	H6B—C6B—C5B	119.13 (8)
C7A—C6A—C5A	121.56 (13)	C7B—C6B—C5B	121.74 (13)
C7A—C6A—H6A	119.22 (8)	C7B—C6B—H6B	119.13 (8)
H7A—C7A—C6A	119.48 (8)	H7B—C7B—C6B	119.56 (8)
C8A—C7A—C6A	121.04 (13)	C8B—C7B—C6B	120.87 (13)
C8A—C7A—H7A	119.48 (9)	C8B—C7B—H7B	119.56 (8)
H8A—C8A—C7A	121.48 (9)	H8B—C8B—C7B	121.36 (8)
C9A—C8A—C7A	117.05 (13)	C9B—C8B—C7B	117.29 (13)
C9A—C8A—H8A	121.48 (8)	C9B—C8B—H8B	121.36 (8)
C4A—C9A—N1A	105.22 (11)	C4B—C9B—N1B	105.43 (11)
C8A—C9A—N1A	131.98 (12)	C8B—C9B—N1B	132.10 (12)
C8A—C9A—C4A	122.81 (12)	C8B—C9B—C4B	122.47 (12)
H10A—N10A—C2A	117.8 (8)	H10B—N10B—C2B	120.3 (8)
C11A—N10A—C2A	124.54 (10)	C11B—N10B—C2B	124.58 (11)
C11A—N10A—H10A	117.3 (8)	C11B—N10B—H10B	115.2 (8)
N10A—C11A—O1A	122.85 (11)	N10B—C11B—O1B	122.75 (12)
C12A—C11A—O1A	122.60 (11)	C12B—C11B—O1B	122.50 (12)
C12A—C11A—N10A	114.55 (11)	C12B—C11B—N10B	114.75 (11)
H12A—C12A—C11A	109.5	H12D—C12B—C11B	109.5
H12B—C12A—C11A	109.5	H12E—C12B—C11B	109.5
H12B—C12A—H12A	109.5	H12E—C12B—H12D	109.5
H12C—C12A—C11A	109.5	H12F—C12B—C11B	109.5
H12C—C12A—H12A	109.5	H12F—C12B—H12D	109.5
H12C—C12A—H12B	109.5	H12F—C12B—H12E	109.5
			
O1A—C11A—N10A—C2A	3.85 (16)	O1B—C11B—N10B—C2B	−1.22 (17)
N1A—C2A—N3A—C4A	0.75 (11)	N1B—C2B—N3B—C4B	−0.30 (12)
N1A—C2A—N10A—C11A	−9.55 (15)	N1B—C2B—N10B—C11B	4.53 (15)
N1A—C9A—C4A—N3A	−0.37 (11)	N1B—C9B—C4B—N3B	−0.84 (11)
N1A—C9A—C4A—C5A	−179.52 (10)	N1B—C9B—C4B—C5B	178.69 (10)
N1A—C9A—C8A—C7A	179.10 (17)	N1B—C9B—C8B—C7B	179.35 (17)
C2A—N1A—C9A—C4A	0.79 (11)	C2B—N1B—C9B—C4B	0.63 (11)
C2A—N1A—C9A—C8A	−179.21 (12)	C2B—N1B—C9B—C8B	−178.87 (11)
C2A—N3A—C4A—C5A	178.81 (11)	C2B—N3B—C4B—C5B	−178.77 (10)
C2A—N3A—C4A—C9A	−0.21 (11)	C2B—N3B—C4B—C9B	0.70 (11)
C2A—N10A—C11A—C12A	−176.65 (13)	C2B—N10B—C11B—C12B	179.08 (14)
N3A—C2A—N1A—C9A	−1.01 (12)	N3B—C2B—N1B—C9B	−0.22 (12)
N3A—C2A—N10A—C11A	170.41 (12)	N3B—C2B—N10B—C11B	−175.67 (12)
N3A—C4A—C5A—C6A	−178.83 (16)	N3B—C4B—C5B—C6B	−178.53 (15)
N3A—C4A—C9A—C8A	179.63 (11)	N3B—C4B—C9B—C8B	178.72 (11)
C4A—N3A—C2A—N10A	−179.22 (9)	C4B—N3B—C2B—N10B	179.88 (9)
C4A—C5A—C6A—C7A	−0.24 (17)	C4B—C5B—C6B—C7B	−0.62 (16)
C4A—C9A—C8A—C7A	−0.90 (16)	C4B—C9B—C8B—C7B	−0.09 (15)
C5A—C4A—C9A—C8A	0.48 (16)	C5B—C4B—C9B—C8B	−1.74 (16)
C5A—C6A—C7A—C8A	−0.20 (19)	C5B—C6B—C7B—C8B	−1.23 (19)
C6A—C5A—C4A—C9A	0.11 (17)	C6B—C5B—C4B—C9B	2.04 (16)
C6A—C7A—C8A—C9A	0.75 (19)	C6B—C7B—C8B—C9B	1.55 (18)
C9A—N1A—C2A—N10A	178.96 (9)	C9B—N1B—C2B—N10B	179.60 (10)

### N-(1H-Benzo[d]imidazol-2-yl)acetamide Hydrogen-bond geometry (Å, º)

**Table:**

*D*—H···*A*	*D*—H	H···*A*	*D*···*A*	*D*—H···*A*
N1*A*—H1*A*···O1*B*	1.03 (1)	2.06 (1)	2.9647 (15)	145 (1)
N1*B*—H1*B*···O1*A*	1.03 (1)	2.05 (1)	2.9693 (15)	148 (1)
N1*A*—H1*A*···O1*A*	1.03 (1)	2.10 (1)	2.6780 (16)	114 (1)
N1*B*—H1*B*···O1*B*	1.03 (1)	2.10 (1)	2.6822 (17)	114 (1)
N10*A*—H10*A*···N3*B*^i^	1.031 (15)	1.948 (15)	2.9757 (16)	174.7 (13)
N10*B*—H10*B*···N3*A*^ii^	1.026 (14)	1.962 (14)	2.9837 (16)	173.7 (11)

Symmetry codes: (i) *x*, *y*+1, *z*; (ii) *x*, *y*−1, *z*.
