# Supplementary material for: Crystal structure and Hirshfeld surface analysis of N-(1H-benzo[d]imidazol-2-yl)acetamide
Source: Acta Crystallogr E Crystallogr Commun. 2026 Apr 29;82(Pt 5):521–4. doi: 10.1107/S2056989026004196 (PMC13148197; doi:10.1107/S2056989026004196)
Supplement: Supplementary file 2 [file e-82-00521-sup2.pdf]

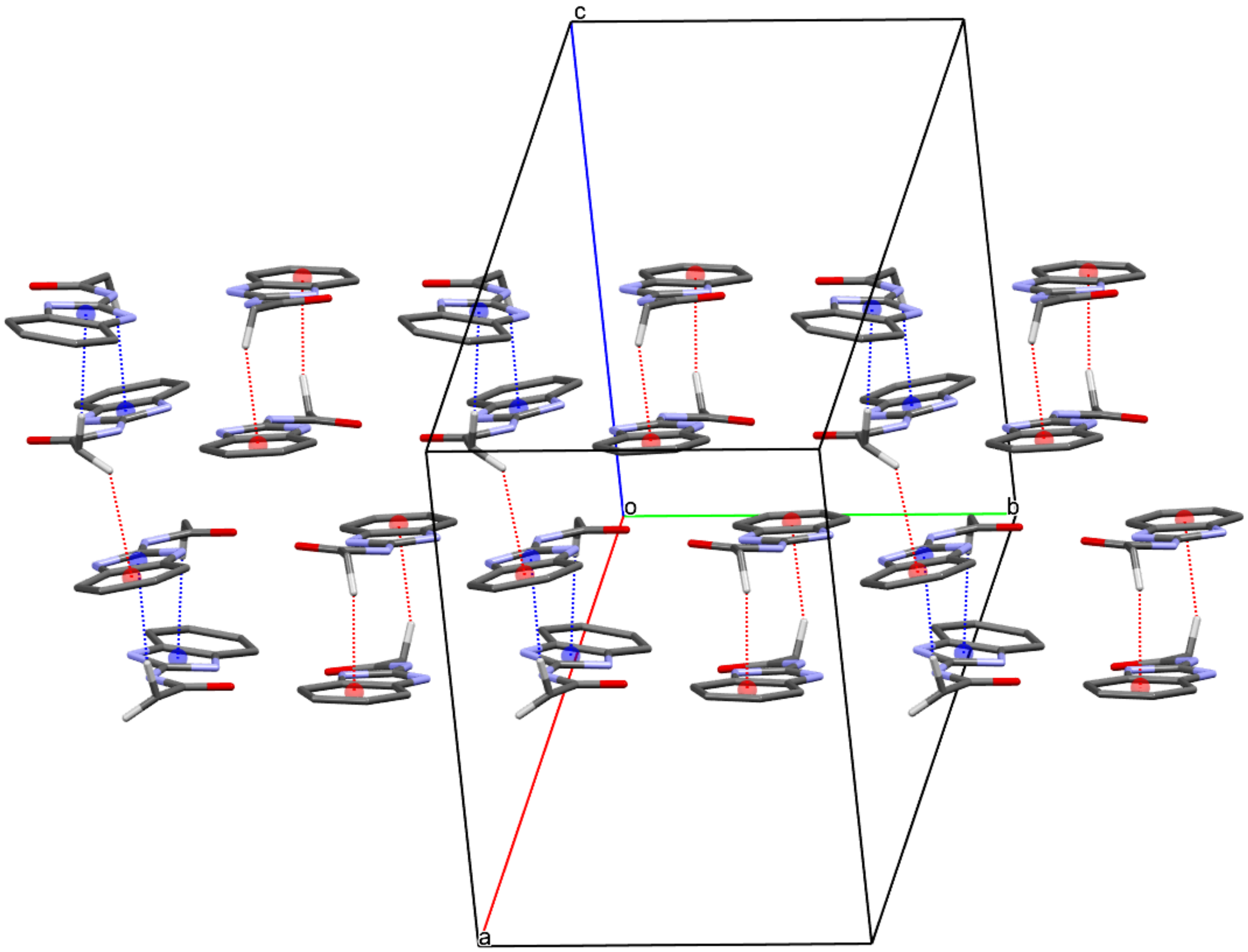

Fig. S1 Dashed red and blue lines denote contacts: C12B—H12B···*Cg*2, C12A—H12F···*Cg*5 and C12B—H12E···*Cg*4, respectively. Red spheres correspond to the ring centroids *Cg*2 and *Cg*4 (benzene rings), and blue spheres to the ring centroids *Cg*5 (five-member hetero rings). H atoms that are not involved in contact are omitted for clarity.

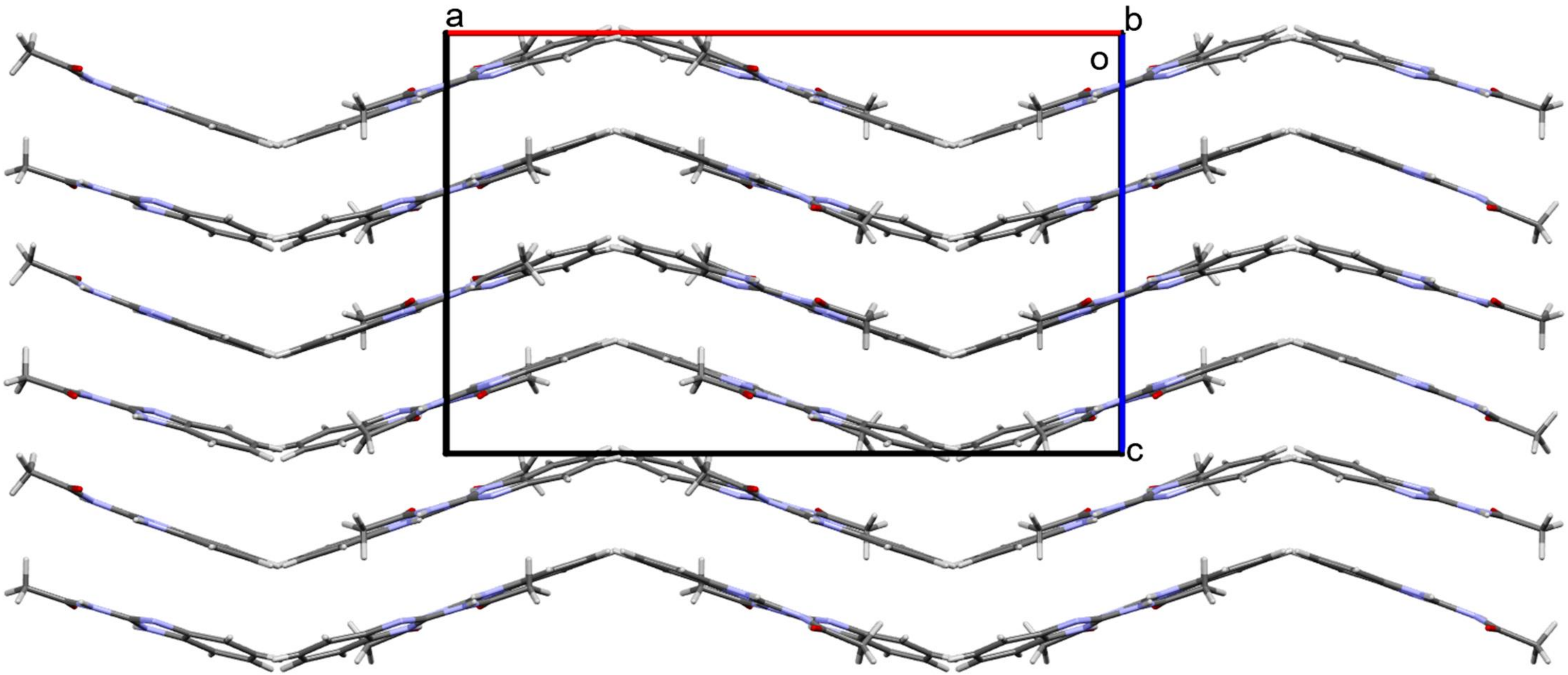

Fig. S2 A view along the crystallographic b axis showing the formation of a herringbone packing motif.

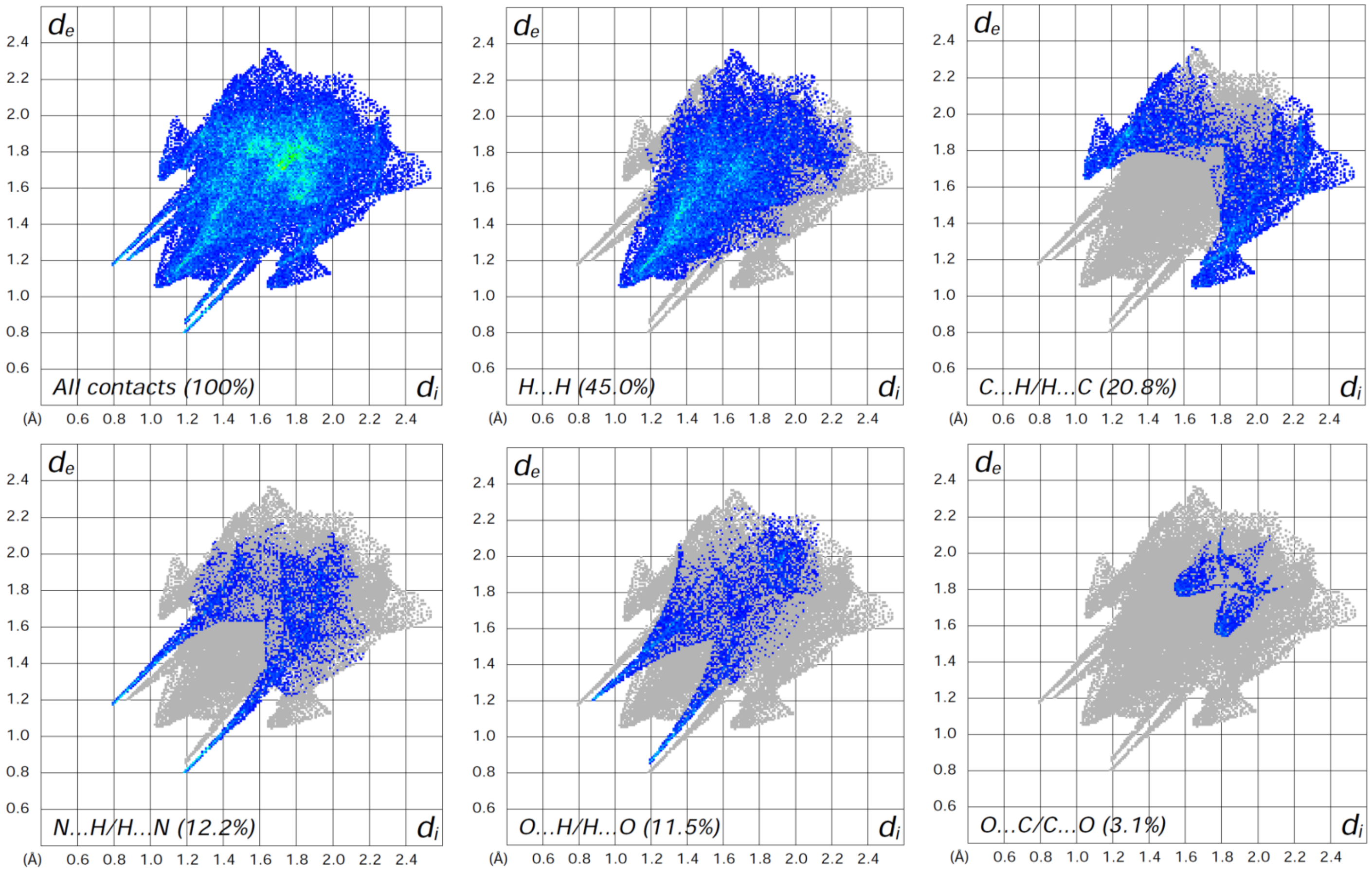

Fig. S3 Two-dimensional fingerprint plots for **1**, showing all interactions (100%), and decomposed into H···H (45%), C···H/H···C (20.8%), O···H/H···O (12.2%), O···H/H···O (11.5%) and O···C/C···O (3.1%) interactions.
